# Cardiac Autonomic Function and Atrial Arrhythmias in Adult Patients With a Univentricular Physiology After Fontan Palliation

**DOI:** 10.1016/j.cjcpc.2025.05.007

**Published:** 2025-06-04

**Authors:** Marieke Nederend, Elizaveta Polyakova, Philippine Kiès, Arie Maan, Sum-Che Man, Marianne Bootsma, Anastasia D. Egorova, Monique R.M. Jongbloed

**Affiliations:** a CAHAL, Center for Congenital Heart Disease Amsterdam Leiden, Leiden University Medical Center, Leiden, the Netherlands; b Department of Cardiology, Leiden University Medical Center, Leiden, the Netherlands; c Department of Anatomy & Embryology, Leiden University Medical Center, Leiden, the Netherlands

**Keywords:** congenital heart disease, Fontan circulation, univentricular heart, heart rate variability, cardiac autonomic nervous system, arrhythmias

## Abstract

**Background:**

Cardiac arrhythmias are frequently encountered in patients with single ventricle physiology palliated by the Fontan circulation. Autonomic dysfunction, an imbalance between sympathetic and parasympathetic systems, is critical in arrhythmogenesis. The relationship between impaired autonomic function, cardiac morphology, ventricular function, and clinical outcomes in adult patients remains poorly defined. This study aims to evaluate cardiac autonomic function using heart rate variability and exercise testing and investigate autonomic factors associated with the prevalence of atrial arrhythmias in adult Fontan patients.

**Methods:**

Consecutive adult Fontan patients were included in this single-centre, cross-sectional study. Cardiac autonomic function was assessed using 24-hour ambulatory electrocardiogram recordings and exercise testing. Factors associated with atrial arrhythmias were evaluated.

**Results:**

Fifty-four patients (median age 22 years, 56% female) were included. Paroxysmal atrial arrhythmias were present in 26% of patients and associated with significantly lower heart rate reserve (60 vs 91 beats per minute [bpm], *P* = 0.004) and chronotropic index (0.46 vs 0.82, *P* = 0.006) during exercise testing. Independent of β-blocker use, the autonomic function parameters, standard deviation of normal-to-normal intervals and standard deviation of the average normal-to-normal intervals calculated of all 5-minute intervals, were reduced in patients with atrial arrhythmias. Ventricular arrhythmias were less frequently encountered (7%).

**Conclusions:**

Atrial arrhythmias, affecting 26% of this cohort, were associated with reduced heart rate reserve and chronotropic index, partly due to β-blocker use, and decreased standard deviation of normal-to-normal intervals and standard deviation of the average normal-to-normal intervals calculated over 5-minute intervals. This suggests impaired autonomic regulation and adaptability in adult Fontan patients, potentially predisposing to late complications. Addressing autonomic dysfunction may help mitigate arrhythmic risks in this population.

## Résumé

**Contexte:**

Les arythmies cardiaques sont fréquentes chez les patients présentant une physiologie univentriculaire palliée par une circulation de Fontan. La dysfonction autonome, un déséquilibre entre les systèmes sympathique et parasympathique, joue un rôle essentiel dans l’arythmogénèse. Le lien entre l’altération de la fonction autonome, la morphologie cardiaque, la fonction ventriculaire et les résultats cliniques chez les patients adultes reste flou. Cette étude vise à évaluer la fonction autonome cardiaque en fonction de la variabilité de la fréquence cardiaque et à l’aide de tests à l’effort, et à déterminer les facteurs autonomes associés à la prévalence des arythmies auriculaires chez les patients adultes ayant subi une intervention de Fontan.

**Méthodologie:**

Des patients adultes consécutifs ayant subi une intervention de Fontan ont été inclus dans cette étude transversale menée dans un seul centre. Leur fonction autonome cardiaque a été évaluée à l’aide d’un électrocardiogramme ambulatoire continu pendant 24 heures et de tests à l’effort. Les facteurs associés aux arythmies auriculaires ont été évalués.

**Résultats:**

Cinquante-quatre patients (âge médian de 22 ans, 56 % de femmes) ont été inclus. Des arythmies auriculaires paroxystiques étaient présentes chez 26 % des patients. Celles-ci étaient associées à une réserve de fréquence cardiaque significativement plus faible (60 *vs* 91 battements par minute [bpm]; *p* = 0,004) et à un indice chronotrope significativement plus bas (0,46 *vs* 0,82; *p* = 0,006) pendant les tests à l’effort. Indépendamment de l’utilisation de β-bloquants, les paramètres de la fonction autonome, l’écart-type des intervalles normaux-à-normaux et l’écart-type de la moyenne des intervalles normaux-à-normaux calculée sur l’ensemble des intervalles de 5 minutes étaient réduits chez les patients atteints d’arythmies auriculaires. Les arythmies ventriculaires étaient moins fréquentes (7 %).

**Conclusions:**

Les arythmies auriculaires, qui concernent 26 % de cette cohorte, ont été associées à une réduction de la réserve de fréquence cardiaque et de l’indice chronotrope, en partie attribuable à l’utilisation d’un β-bloquant, ainsi qu’à une diminution de l’écart-type des intervalles normaux-à-normaux et de l’écart-type de la moyenne des intervalles normaux-à-normaux calculée sur des intervalles de 5 minutes. Ces observations indiquent une altération de la régulation autonome et une adaptabilité chez les patients adultes ayant subi une circulation de Fontan, ce qui pourrait les prédisposer à des complications tardives. La prise en charge de la dysfonction autonome peut contribuer à atténuer les risques associés aux arythmies dans cette population.

Although the Fontan operation has significantly improved survival in patients with single ventricle physiology, the growing population of adult Fontan patients faces high rates of long-term complications, with only 41% free of serious events by age 40 years.^1^, ^2^, ^3^, ^4^, ^5^, ^6^, ^7^, ^8^, ^9^, ^10^

Cardiac arrhythmias are among the most significant complications in this population, contributing to morbidity and mortality, including sudden cardiac death. The autonomic nervous system modulates cardiac rhythm and regulates adaptive responses to physiological demands. Although complex, parasympathetic activity is generally cardioprotective, whereas sympathetic overactivity is linked to an adverse outcome, such as ventricular and atrial arrhythmias, heart failure, and mortality.^11^, ^12^, ^13^ Autonomic dysfunction, a disbalance between the sympathetic and parasympathetic branches of the autonomic nervous system, plays a key role in arrhythmogenesis and may be a common denominator of late complications in Fontan patients.^14^, ^15^, ^16^, ^17^ Autonomic dysfunction impacts electrophysiological parameters such as action potential duration, effective refractory period, QT dispersion, and the threshold for ventricular fibrillation, thereby contributing to arrhythmogenic mechanisms such as abnormal automaticity, triggered activity, and re-entry.^17^

Cardiac autonomic nervous activity (CANA) can be assessed noninvasively through heart rate variability (HRV), heart rate recovery, QT interval variability, T-wave alternans, baroreflex sensitivity, skin conductance, positron emission tomography, and single-photon emission computed tomography, or invasively using pharmacologic testing. HRV, a widely used noninvasive marker, reflects both parasympathetic and sympathetic activity.^18^ It can be analysed using frequency domains (the distribution of power [variance] of HRV across different frequency bands, providing insights into specific components of autonomic regulation: eg, low-frequency [LF] power) and time domains (involves examining the intervals between consecutive heartbeats [R-R intervals] over time: eg, standard deviation [SD] of normal-to-normal [NN] intervals [SDNN]), each providing distinct insights into autonomic balance.^18^

Autonomic dysfunction has been observed in up to 50% of Fontan patients.^14^^,^^19^^,^^20^ Studies in paediatric patients suggest that Fontan patients exhibit reduced HRV compared with healthy controls, with evidence of progressive autonomic impairment over time and cumulative dysfunction across successive surgical palliation stages.^15^ Abnormal values of clinical markers of CANA have been associated with arrhythmias and other complications in patients with complex congenital heart disease (CHD).^21^^,^^22^ Despite the high incidence of arrhythmias in Fontan patients, the relationship between impaired CANA, cardiac morphology, ventricular function, and clinical outcomes in adults remains poorly understood.^14^^,^^19^^,^^20^^,^^23^, ^24^, ^25^

This study therefore aims to (1) report on a contemporary real-world cohort of adult patients with a Fontan circulation and evaluate their cardiac autonomic function using HRV and exercise testing; and (2) investigate autonomic functional factors associated with the presence of atrial arrhythmias in this population.

## Methods

This single-centre, cross-sectional cohort study was performed at the Department of Cardiology, Center for Congenital Heart Disease Amsterdam Leiden, Leiden University Medical Center.

### Data collection and outcomes

All consecutive adult Fontan patients under follow-up at our institution as of July 2023 were included. Data on morphologic substrate and clinical and demographic data were collected from electronic patient records. All reported data were obtained as part of routine clinical care. Data from the most recent outpatient clinic visit were collected, including medical history (eg, hospital admissions and/or emergency department visits and late sequelae), symptoms, pharmacologic therapy, physical examination, electrocardiogram (ECG), 24-hour ambulatory ECG recording, transthoracic echocardiography, magnetic resonance imaging, laboratory investigations, and exercise testing. Fontan conduit size, in case of cavopulmonary connection, was registered from the operative report. Clinical investigations up to 2 years before the most recent follow-up moment were included; magnetic resonance imaging was included up to 5 years before the most recent follow-up. Echocardiograms were performed with commercially available ultrasound systems and were analysed in EchoPAC (GE Medical Systems, IL) by cardiologists with dedicated expertise in congenital imaging. Fontan-associated liver disease was defined as any form of liver fibrosis, cirrhosis, or hepatocellular carcinoma at dedicated imaging.^5^ Cyanosis at rest was defined as a transcutaneous oxygen saturation <93%. Thrombosis, including ischemic cerebrovascular accident, transient ischemic attack, or pulmonary embolism, was diagnosed based on clinical presentation, specialist evaluation, and imaging of the brain or lungs. The presence of venovenous collaterals was diagnosed by means of a computed tomography scan and/or catheterization procedure.

### Cardiac autonomic function parameters

Cardiac autonomic function was evaluated through HRV on 24-hour ambulatory ECG recording analyses and exercise test assessment.

### 24-hour ambulatory ECG recording analyses

**Figure 1:**
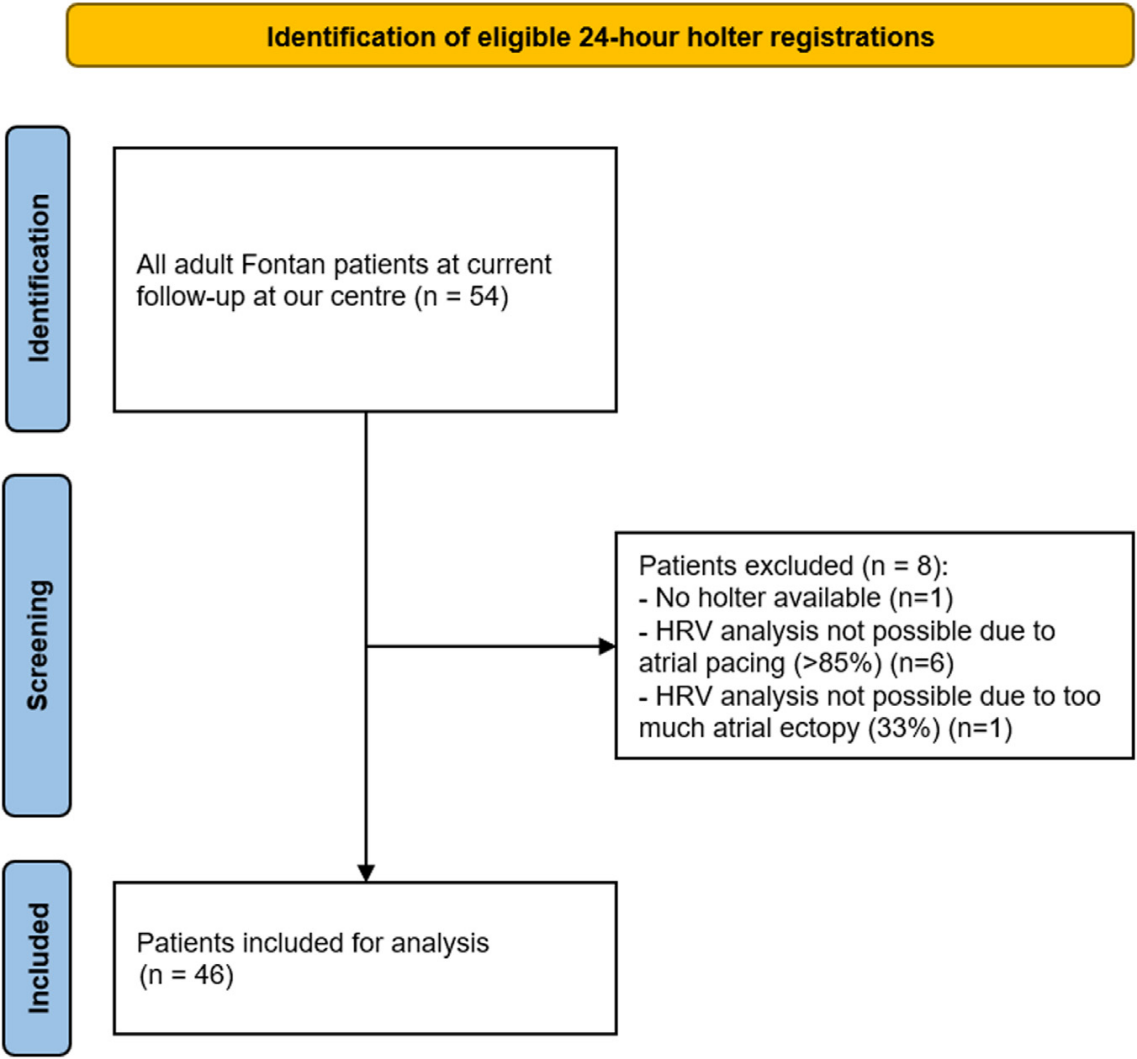
Flow chart for the screening and selection of patients with univentricular physiology after Fontan palliation and eligible 24-hour ambulatory electrocardiogram recording registrations. Fontan patients, patients with univentricular physiology after Fontan palliation; HRV, heart rate variability.

Patients who had an eligible 24-hour ambulatory ECG recording performed at adult age were selected (Fig. 1). For each patient, the most recent available and technically satisfactory 24-hour ambulatory ECG recording registration was analysed as previously described.^21^ The registration was included if more than 18 hours of registration, including the complete night, was available and if there was <10% of ectopy.^26^ Patients with >85% atrial pacing were excluded. Analyses of the 24-hour ambulatory ECG recording and the HRV analysis were performed with CardioDay Holter ECG Software (GE Healthcare). Beats were automatically classified as normal, supraventricular, ventricular, artefact, or of uncertain origin. Junctional and atrial rhythms were classified as normal. These classifications were manually validated and corrected if needed. Sinus rhythm was defined as a positive p-wave in 24-hour ambulatory ECG recording channels II and III. The 24-hour ambulatory ECG recording–based measures of cardiac autonomic function are depicted in Table 1. HRV analysis included the following variables for time domain variables: SDNN, SD of the average NN intervals calculated of all 5-minute intervals (SDANN), percentage of adjacent NN intervals that differ by>50 milliseconds (ms), and square root of the mean squared differences of successive NN intervals. NN intervals refer to the time between successive normal heartbeats, specifically excluding abnormal beats (like ectopic beats or arrhythmias). In addition, frequency domain variables were calculated in 5-minute intervals: very LF power (0.003-0.04 Hz), LF power (0.04-0.15 Hz), high-frequency (HF) power (0.15-0.4 Hz), the LF/HF ratio, and the total power. For the QT variability measurements, channel III was selected, as the most representative, stable lead. Channel III reflects lead V5 on the 12-lead ECG. The QT interval corrected according to Bazett ($\text{QTc}=\frac{\text{QT}}{\surd \text{RR}}$(QTc) where RR is the interval between consecutive R waves) was calculated at 5-minute intervals. The mean QTc interval was calculated for the hours of the recording, with its SD.

**Table 1:** Twenty-Four-hour ambulatory electrocardiogram recording–based measurements, including heart rate variability, and QT-variability

Abbreviation	Principle and/or calculation	Autonomic Nervous System Association
SDNN (ms)	Standard deviation of all NN intervals	Parasympathetic and sympathetic nervous system activity
SDANN (ms)	Standard deviation of the average NN intervals calculated over 5-minute intervals	Parasympathetic and sympathetic nervous system activity
pNN50 (%)	Percentage of adjacent NN intervals that differ by >50 ms	Parasympathetic nervous system activity
rMSSD (ms)	Square root of the mean squared differences of successive NN intervals	Parasympathetic nervous system activity
VLF (ms^2^)	Very low-frequency power (0.003-0.04 Hz)	Sympathetic nervous system activity
LF (ms^2^)	Low-frequency power (0.04-0.15 Hz)	Parasympathetic and sympathetic nervous system activity
HF (ms^2^)	High-frequency power (0.15-0.4 Hz)	Parasympathetic nervous system activity
LF/HF ratio	Ratio between low-frequency power and high-frequency power	Sympathetic/parasympathetic balance
Total power (ms^2^)	The total power	Parasympathetic and sympathetic nervous system activity
Mean QTc	Mean corrected QT interval according to Bazett, calculated over 5-minute intervals	
SD QT	The standard deviation of the mean corrected QT interval according to Bazett, calculated over 5-minute intervals	

NN, normal-to-normal.

### Exercise test

Patients underwent a symptom-limited exercise test on a bicycle ergometer, following a protocol with 1-minute stages, and incremental workload increases until exhaustion. Throughout the test, a 12-lead ECG and pulse oximeter were monitored, and a breath-by-breath analysis of expired air was used to measure oxygen consumption (VO_2_). The age-predicted maximal heart rate was calculated using the formula (208 − 0.7 × age),^27^ and the chronotropic index was derived as follows: ([maximal heart rate − resting heart rate ]/[age-predicted maximal heart rate − resting heart rate]).^28^ Heart rate recovery was defined as the rate of heart rate decline during the first minute of active recovery.

### Arrhythmias

Patients were screened for the presence of supraventricular and/or ventricular arrhythmias. Medical history was reviewed, and arrhythmias were documented if identified through a 12-lead ECG, 24-hour ambulatory ECG recording, bicycle ergometry, cardiac implantable device data, or records from another centre.

### Ethics statement

All tests and procedures performed involving human participants were in accordance with the ethical standards of the institutional and/or national research committee and with the 2013 Declaration of Helsinki or comparable ethical standards. Appropriate local scientific board approval was obtained, and the need for written informed consent was waived by the institutional medical ethical board (protocol number: 2023-004). All patients provided consent for registration of their data and publication.

### Statistical analysis

All statistical analyses were performed in IBM SPSS Statistics for Windows, version 29.0 (IBM Corp). Normally distributed continuous data are displayed as mean ± SD, and non-normally distributed continuous data are displayed as median with the first and third quartile [Q1-Q3]. Proportions are displayed as numbers (percentages). Normality was graphically assessed and additionally tested with the use of the Shapiro-Wilk test. In the univariate analyses comparing cardiac autonomic function parameters in patients with and without arrhythmias, an unpaired *t* test or a Mann-Whitney *U* test was used as appropriate for the comparison of continuous data between different subgroups. For categorical data, the χ^2^ test was used. A *P* value of <0.05 was considered statistically significant.

## Results

### Fontan population

A total of 54 adult Fontan patients were identified. The median age at last follow-up was 22 [20-27] years; 30 patients (56%) were female. A detailed overview of the anatomy is given in Table 2. Twenty-seven patients (50%) had a systemic left ventricle as the dominant ventricle supporting the systemic circulation, 21 (39%) had a systemic right ventricle, and 6 (11%) had biventricular contribution to the systemic circulation as a functional single ventricle. The median age at the Fontan operation was 3.5 [3-5] years; 43 patients (80%) had an extracardiac conduit, 6 (11%) an atriopulmonary connection, and 5 (9%) an intracardiac/lateral tunnel. The median Fontan (extracardiac) conduit size was 16 [16-16] mm.

**Table 2:** Patient demographics, anatomy, and medical history

Characteristic	All Fontan patients (n = 54)
Patient demographics	
Age at last follow-up (y)	22 [20-27]
Female	30 (56)
Anatomy and history	
Diagnosis at birth	
AVSD	6 (11)
ccTGA	5 (9)
Crisscross heart	1 (2)
Double inlet LV	9 (17)
Double outlet RV	7 (13)
Hypoplastic left heart/mitral atresia	11 (20)
Pulmonary stenosis/atresia	5 (9)
Tricuspid atresia	9 (17)
Truncus arteriosus	1 (2)
Dominant ventricle for systemic circulation	
LV	27 (50)
RV	21 (39)
Biventricular	6 (11)
Age at Fontan (y)	3.5 [3-5]
Fontan type	
Atriopulmonary connection	6 (11)
Intracardiac/lateral tunnel	5 (9)
Extracardiac conduit	43 (80)
Fontan conduit size (mm)∗	16 [16-16]
Patent fenestration	6 (11)
Cardiac implantable electronic device	8 (15)
Type of device†	
AAI-PM	3 (37)
DDD-PM	5 (63)

All data are shown as median [Q1-Q3] or n (%).

AAI-PM, atrial pacemaker; AVSD, atrioventricular septal defect; ccTGA, congenitally corrected transposition of the great arteries; DDD-PM, dual-chamber pacemaker; LV, left ventricle; Q, quartile; RV, right ventricle; SD, standard deviation.

∗ In case of cavopulmonary connection.

† Percentages are calculated within preceding subgroup, not the total population: 5/8 for type of device.

The clinical and functional findings at the most recent follow-up are shown in Table 3. Estimated systemic systolic ventricular function on transthoracic echocardiography was relatively well preserved, with only 4 patients (8%) having moderately reduced function. Haemodynamically significant atrioventricular-valve regurgitation was present in 3 patients (6%). Comparably, on magnetic resonance imaging, available in 33 (61%) patients, the mean ejection fraction was 47% ± 7% and the estimated cardiac output 5.4 ± 1.7 L/min. Blood count, renal function, and serum N-terminal prohormone of brain natriuretic peptide levels were all within normal ranges. Fourteen patients (26%) had documented arrhythmias, where the majority (93%) had supraventricular arrhythmias and 1 patient (7%) had both supraventricular and (nonsustained) ventricular arrhythmias (Table 4). Twenty-four patients (44%) had a hospital admission and/or an unplanned visit in the past 5 years, accounting for a total of 39 events. Arrhythmias were the most frequently encountered reason for an (unplanned) visit accounting for 15 events (38% of total presentations). Other frequent causes included dyspnoea (unrelated to heart failure), fever, syncope (non-arrhythmogenic), and chest discomfort, each accounting for 4 or fewer events.

**Table 3:** Clinical and functional findings at last follow-up

Clinical and functional findings	All Fontan patients (n = 54)
Physical examination	
Systolic blood pressure (mm Hg)	124 ± 12
BMI (kg/m^2^)	23.0 ± 3.6
Pharmacologic therapy	
Acetylsalicylic acid	18 (33)
Anticoagulation	33 (61)
Vitamin K antagonist	27 (50)
DOAC	6 (11)
β-Blocker	7 (13)
ACEi/ARB/ARNI	9 (17)
SGLT2i	1 (2)
MRA	3 (6)
Diuretics	5 (9)
PDE5-inhibitors	2 (4)
ECG	
QRS duration (ms)	115 ± 20
Imaging	
Systemic ventricle function on TTE	
Good	12 (22)
Mildly reduced	38 (70)
Moderately reduced	4 (8)
Systemic ventricle ejection fraction on MRI (%)	47 ± 7
Cardiac output on MRI (L/min)	5.4 ± 1.7
AV-valve regurgitation on TTE	
Grade 1 or less	32 (59)
Grade 2	18 (33)
Grade 3	3 (6)
Indeterminate	1 (2)
VA-valve regurgitation on TTE	
Grade 1 or less	43 (80)
Grade 2	8 (15)
Grade 3 or more	2 (4)
Indeterminate	1 (2)
Laboratory findings	
Hb (mmol/L/g/dL)	9.5 ± 1/15.3 ± 1.6
eGFR (mL/min/1.73 m^2^)	114 ± 16
NT-proBNP (pg/mL)	160 ± 142

All data are shown as mean ± SD or n (%).

ACEi, angiotensin-converting enzyme inhibitor; ARB, angiotensin receptor blocker; ARNI, angiotensin receptor-neprilysin inhibitor; AV, atrioventricular; BMI, body mass index; DOAC, direct oral anticoagulants; ECG, electrocardiogram; eGFR, estimated glomerular filtration rate; Hb, hemoglobin; MRA, mineralocorticoid receptor antagonist; MRI, magnetic resonance imaging; NT-proBNP, N-terminal prohormone brain natriuretic peptide (reference value <161 ng/L for male and <247.0 ng/L for female); PDE5, phosphodiesterase type 5; SD, standard deviation; SGLT2i, sodium-glucose cotransporter-2 inhibitors; TTE, transthoracic echocardiography; VA, ventriculoarterial.

**Table 4:** Clinical outcomes and late sequelae affecting multiple organ systems

Clinical outcomes and late sequelae	All Fontan patients (n = 54)
Hospital presentations and/or admissions	24 (44)
Total number	39
Reason for presentation/admission	
Arrhythmia∗	15 (38)
Heart failure∗	1 (3)
Other∗	23 (59)
Arrhythmia	14 (26)
Supraventricular	13 (93)
Both supraventricular and ventricular	1 (7)
Ablation	8 (57)
Cyanosis at rest	4 (8)
Fontan-associated liver disease	15 (28)
Protein losing enteropathy	2 (4)
Thrombosis	6 (11)
Ischemic CVA or TIA	5 (9)
Pulmonary embolus	2 (4)
Venovenous collaterals	24 (44)

All data are shown as n (%) from the total population (n = 54), unless indicated with an asterisk.

CVA, cerebrovascular accident, TIA, transient ischemic attack.

∗ Percentages are calculated within total number of admissions (n = 39).

### Cardiac autonomic function parameters

Patients were screened for eligible 24-hour ambulatory ECG recording registrations, and a total of 46 (85%) patients were included for analysis (Fig. 1). An exercise test was available in 47 (87%) patients. Cardiac autonomic function parameters (24-hour ambulatory ECG recording registration findings and exercise test) are shown in Table 5.

**Table 5:** Findings on noninvasive cardiac autonomic nervous activity parameters in Fontan patients with and without arrhythmias, last column with the exclusion of patients using β-blockers

	All Fontan patients (n = 46)	Patients with arrhythmia (n = 10)	Patients without arrhythmia (n = 36)	*P* value	*P* value (exclusion of β-blockers, n = 41)
*24-hour ambulatory ECG recording parameters*					
Dominant rhythm					
Sinus	43 (94)	8 (80)	35 (97)	0.067	0.204
Atrial	1 (2)	1 (10)	–	–	–
Junctional	2 (4)	1 (10)	1 (3)	0.364	0.204
Heart rate variability					
SDNN (ms)	171 ± 78	129 ± 45	182 ± 81	0.056	**0.033**
SDANN (ms)	147 ± 65	112 ± 43	157 ± 67	**0.048**	**0.031**
pNN50 (%)	16 [4-35]	12 [4-18]	18 [4-38]	0.324	0.182
rMSSD (ms)	53 [30-102]	44 [33-74]	59 [25-107]	0.567	0.218
VLF (ms^2^)	778 [293-2368]	1117 [201-2535]	580 [322-2344]	0.567	0.822
LF (ms^2^)	219 [73-754]	140 [66-1064]	244 [78-824]	0.780	0.931
HF (ms^2^)	47 [11-317]	30 [11-497]	48 [10-354]	0.926	0.876
LF/HF ratio	3.4 [2.3-7.6]	2.6 [0.9-7.3]	3.8 [2.6-7.8]	0.258	0.323
Total power (ms^2^)	1425 [562-5097]	1521 [947-7972]	1315 [504-5091]	0.576	0.628
QT variability					
Mean QTc	460 ± 20	454 ± 19	461 ± 20	0.279	0.943
SD QT	14 ± 5	14 ± 6	15 ± 5	0.819	0.319
*Cardiopulmonary exercise test*					
Maximal exercise capacity (W)	134 ± 47	132 ± 40	135 ± 50	0.855	0.954
Percent of predicted exercise capacity (W) (%)	69 ± 19	67 ± 15	70 ± 21	0.663	0.842
VO_2_max (mL/kg/min)	22.4 ± 5.9	21.5 ± 5.0	22.6 ± 6.2	0.585	0.801
Percent of predicted VO_2_max (%)	55 ± 13	53 ± 13	56 ± 14	0.606	0.799
Percent of predicted maximal heart rate (%)	82 ± 16	70 ± 17	86 ± 14	**0.002**	0.059
Heart rate reserve (bpm)	84 ± 33	60 ± 32	91 ± 30	**0.004**	0.063
Heart rate recovery (bpm)	21 ± 10	19 ± 16	21 ± 8	0.600	0.767
Chronotropic index	0.74 [0.48-0.85]	0.46 [0.34-0.74]	0.82 [0.68-0.88]	**0.006**	0.099
Oxygen pulse (mL/beat)	9.4 [7.9-11.1]	11.9 [8.2-15.7]	9.3 [7.5-10.2]	0.078	0.467

All data are shown as median [Q1-Q3], mean ± SD, or n (%). Bold *p*-values are statistically significant.

ECG, electrocardiogram; HF, high frequency power; LF, low frequency power; NN, normal-to-normal; pNN50, percentage of adjacent NN intervals that differ by>50 ms; Q, quartile; QTc, corrected QT interval according to Bazett; rMSSD, square root of the mean squared differences of successive NN intervals; SD, standard deviation; SDANN, standard deviation of the average NN intervals calculated over 5-minute intervals; SDNN, standard deviation of all NN intervals; VLF, very low frequency power; VO_2_max, maximal oxygen consumption.

The most prevalent rhythm during the registration was sinus rhythm in the majority of patients (43 patients, 94%), 2 patients (4%) had junctional rhythm, and 1 patient (2%) had an atrial rhythm. The mean SDNN was 171 ± 78 ms, and the SDANN was 147 ± 65 ms. The median total power was 1425 [562-5097] ms^2^, and the median LF/HF power ratio was 3.4 [2.3-7.6]. Exercise capacity was reduced with a mean percent of predicted exercise capacity (watt) of 69% ± 19% and percent of predicted peak VO_2_ was 55% ± 13%, with an impaired chronotropic index (reference value >0.8)^28^ and percent of predicted maximal heart rate of 82% ± 16%. The heart rate recovery was 21 ± 10 bpm.

### Cardiac autonomic function parameters in patients with arrhythmia and without arrhythmia

Comparison of cardiac autonomic function parameters in patients with arrhythmia and without arrhythmia can be found in Table 5. At exercise testing, those with atrial arrhythmias had lower heart rate reserve and chronotropic index (60 vs 91 bpm, *P* = 0.004, and 0.46 vs 0.82, *P* = 0.006, respectively). However, this effect was no longer significant when corrected for the use of β-blockers **(**Table 5 and Supplemental Table S1). In 24-hour ambulatory ECG recording registrations, the SDANN was significantly lower in Fontan patients with atrial arrhythmias, whereas the other HRV parameters did not differ between those with and without atrial arrhythmias. The difference in SDANN was independent of the use of β-blockers, as was SDNN (Table 5 and Supplemental Table S1).

## Discussion

The main findings of this study are that (1) atrial arrhythmias are common in Fontan patients, with a reported prevalence of 26% in the present cohort, whereas ventricular arrhythmias have a lower prevalence (7%). (2) Patients with atrial arrhythmias have a lower heart rate reserve and chronotropic index, which is at least partly attributable to β-blocker use, and, independent of β-blocker use, lower SDNN and SDANN. Low SDNN and SDANN values indicate autonomic dysfunction and impaired autonomic regulation, respectively.^29^ These data are in line with decreased autonomic adaptability and regulation in this patient group, which may predispose patients for late complications after Fontan palliation.

### Findings in relation to literature

To our knowledge, this is the first study to specifically examine autonomic function in relation to arrhythmias in adult patients with a Fontan circulation, contributing to bridging a critical gap in currently existing literature. In literature, HRV and/or other CANA parameters are significantly reduced in Fontan patients as compared with healthy controls.^14^, ^15^, ^16^^,^^24^^,^^25^^,^^30^, ^31^, ^32^, ^33^, ^34^, ^35^, ^36^, ^37^, ^38^, ^39^, ^40^, ^41^, ^42^, ^43^, ^44^, ^45^ Most studies focused on paediatric Fontan patients,^15^^,^^16^^,^^24^^,^^25^^,^^30^, ^31^, ^32^, ^33^, ^34^^,^^37^^,^^39^, ^40^, ^41^, ^42^, ^43^^,^^46^ with only few reporting on adult Fontan patients.^14^^,^^35^^,^^36^^,^^38^^,^^44^^,^^45^ Several studies reported on a possible relationship with clinical outcomes.^14^^,^^24^^,^^25^^,^^36^^,^^41^^,^^44^^,^^45^ Okolska et al.^44^ found a correlation between significantly impaired HRV and age at the time of surgical intervention, the time since operation, reduced exercise capacity, and end-organ complications in terms of γ-glutamyl transpeptidase levels. In addition, Davos et al.^14^ noted that stronger baroreflexes were associated with higher rates of sustained atrial tachyarrhythmia in Fontan patients. In CHD, including but not limited to Fontan patients, reduced HRV had prognostic implications for mortality and survived sudden cardiac death in adults, and reduced parasympathetic activity was linked with diminished exercise capacity.^36^^,^^45^ Conversely, Ohuchi et al.^24^^,^^25^^,^^41^ reported that CANA parameters did not correlate with functional classification, symptoms, neurohormonal activity, or haemodynamic variables, nor could they predict for future clinical events.

Studies in adult patients report more pronounces autonomic dysfunction linked to arrhythmia risk, reduced exercise capacity, and increased mortality risk.^14^^,^^35^^,^^36^^,^^38^^,^^44^^,^^45^ As autonomic dysfunction in Fontan patients is a potentially progressive process, in our relatively young cohort of adult Fontan patients with a fairly preserved systolic function, autonomic dysfunction might precede clinically overt heart failure. Repeated measurements over time could lead to more pronounced results.

### Cardiac autonomic dysfunction in Fontan failure—potential etiologies

There are multiple explanations for impaired CANA in Fontan patients: (1) intrinsically related to CHD, (2) effects of surgical correction, and (3) attributable to heart failure. First, autonomic dysfunction might be intrinsically related to CHD, for example, by a deficient contribution of neural crest cells during development,^47^ predisposing for heart failure later in life. Evidence of early impaired CANA is seen in foetuses and infants with CHD where HRV is already reduced.^48^, ^49^, ^50^ Secondly, Fontan surgery itself can lead to direct or indirect damage, such as ischemia or denervation, as is reported for specific subgroups of patients with CHD and supported by the progressive impairment across the stages of the Fontan operation.^20^^,^^33^^,^^39^^,^^51^, ^52^, ^53^ Lastly, autonomic dysfunction may be an early sign of subclinical myocardial dysfunction, which can be further exacerbated during progression to overt heart failure.^47^ This is supported by non-CHD where disbalance in CANA is generally attributed to be a result of heart failure, further deteriorating its clinical course.^11^^,^^54^ Whether autonomic dysfunction is a cause or consequence of heart failure remains a question in the complex interplay between autonomic dysfunction, (subclinical) heart failure, and arrhythmias in Fontan patients.

### Fontan surgery and sinus node dysfunction—is HRV an adequate marker?

Sinus node dysfunction (SND) is a common late complication in Fontan patients^8^ and may predispose atrial arrhythmias.^55^ Dahlqvist et al.^34^ explored HRV in Fontan patients with and without SND, including those requiring pacemakers. In patients with a pacemaker, the preimplantation 24-hour ECG recordings showed a tendency toward decreased HRV compared with Fontan patients with SND without pacemaker treatment, suggesting that HRV trends could help monitor worsening SND.^34^ In addition, serial ECGs may also reveal progressive autonomic imbalance and bradycardia.^43^ Conversely, Davos et al.^14^ found stronger baroreflexes linked to more arrhythmias, arguing against SND as the leading mechanism as SND would likely reduce the ability to respond to autonomic modulation. In our study, we could not analyse HRV by SND or pacemaker status, but did observe more pacemakers in the arrhythmia group (29% vs 10%) and lower SDNN and SDANN values, regardless of β-blocker use.

### Gaps in literature

Although considerable research has been performed on HRV in Fontan patients, current gaps in literature include the relative scarcity of longitudinal data in adult-only patient cohorts. In addition, data on the influence of lifestyle and sex, anatomy and specific “driving” factors for the development of cardiac autonomic dysfunction, and definite results of the relation with clinical outcomes within specific groups of Fontan patients are still scarce.

### Limitations

The study has several limitations inherent to its nature. First, the small and heterogeneous study population is reflective of the rarity of the Fontan population. The single-centre design and small cohort size precluded the feasibility of multivariate analyses. The risk of type I error due to multiple comparisons should be recognized. While minimizing a type II error (incorrectly rejecting an association that is in fact present) was prioritized in this explorative analysis, we acknowledge that the lack of formal adjustment may increase the likelihood of detecting associations that in fact occur by chance. Among the 54 Fontan patients included, only 46 had 24-hour ambulatory ECG recordings, and 47 had exercise tests suitable for analysis, resulting in the primary outcome, CANA, being available for only 85%-87% of the cohort. In addition, the study lacks a (matched) control group. However, the pronounced autonomic dysfunction in Fontan patients has previously been widely documented and validated against control groups. Importantly, this study focuses on evaluating CANA in a real-world, contemporary cohort—a less commonly explored area in the literature. This is particularly valuable, as much of the published research either focuses exclusively on paediatric patients or combines various CHD populations, which dilutes the specificity of findings for adult Fontan patients. Another limitation of our study is the potential bias introduced by β-blocker use. β-Blockers are known to influence heart rate response to exercise and significantly affect HRV, increasing vagal tone and reducing sympathetic activation. Although β-blockers may have contributed to observed HRV differences, the lower SDNN and SDANN values in patients with arrhythmias remained significant, independent of β-blocker use.

## Conclusions

Atrial arrhythmias are common in Fontan patients, with a reported prevalence of 26% in the present cohort. There was a relatively low prevalence of ventricular arrhythmias (7%). Patients with atrial arrhythmias had a lower heart rate reserve and chronotropic index, which was at least partly attributable to β-blocker use, and, independent of β-blocker use, lower SDNN and SDANN. These data indicate decreased autonomic adaptability and regulation in this patient group, which may predispose patients for late complications after Fontan palliation.
