# Supplementary material for: Cardiac Autonomic Function and Atrial Arrhythmias in Adult Patients With a Univentricular Physiology After Fontan Palliation
Source: CJC Pediatr Congenit Heart Dis. 2025 Jun 4;5(1):23–31. doi: 10.1016/j.cjcpc.2025.05.007 (PMC12946902; doi:10.1016/j.cjcpc.2025.05.007)
Supplement: Supplementary Material [file mmc1.docx]

**Supplementary Material**

**Supplemental Table S1**

Findings on non-invasive cardiac autonomic nervous activity parameters in Fontan patients with and without arrhythmias, with exclusion of patients using beta-blockers.

**Supplemental Table S1:** Findings on non-invasive cardiac autonomic nervous activity parameters in Fontan patients with and without arrhythmia’s, with exclusion of patients using beta-blockers

|  | **All Fontan patients (n=41)** | **Patients with arrhythmia (n=7)** | **Patients without arrhythmia (n=34)** | **p-value** |
| --- | --- | --- | --- | --- |
| **24-hour ambulatory ECG recording parameters** | | | | |
| Dominant rhythm |  |  |  |  |
| Sinus | 39 (95%) | 6 (86%) | 33 (97%) | 0.204 |
| Junctional | 2 (4%) | 1 (14%) | 1 (3%) | 0.204 |
| **Heart rate variability** |  |  |  |  |
| SDNN, ms | 177 ± 78 | 120 ± 41 | 189 ± 80 | **0.033** |
| SDANN, ms | 153 ± 66 | 104 ± 45 | 163 ± 66 | **0.031** |
| pNN50, % | 16 [5-38] | 8 [3-16] | 20 [6-38] | 0.182 |
| rMSSD, ms | 53 [32-102] | 34 [31-53] | 62 [30-114] | 0.218 |
| VLF, ms^2^ | 787 [342-2380] | 1026 [209-2357] | 709 [350-2484] | 0.822 |
| LF, ms^2^ | 260 [88-927] | 114 [74-2106] | 296 [93-898] | 0.931 |
| HF, ms^2^ | 48 [13-383] | 34 [18-1098] | 50 [12-377] | 0.876 |
| LF/HF ratio | 3.3 [2.3-6.1] | 2.8 [1.0-4.0] | 3.6 [2.5-6.5] | 0.323 |
| Total power, ms^2^ | 1442 [670-5102] | 1442 [1142-7675] | 1414 [595-5097] | 0.628 |
| **QT variability** |  |  |  |  |
| Mean QTc | 461 ± 19 | 461 ± 15 | 461 ± 20 | 0.943 |
| SD QT | 14 ± 5 | 13 ± 6 | 15 ± 5 | 0.319 |
| **Cardiopulmonary exercise test** | | | | |
| Maximal exercise capacity, watt | 137 ± 49 | 136 ± 40 | 137 ± 51 | 0.954 |
| Percent of predicted exercise capacity (watt), % | 71 ± 19 | 69 ± 13 | 71 ± 20 | 0.842 |
| VO_2_max, mL/kg/min | 22.9 ± 6.1 | 23.5 ± 5.3 | 22.7 ± 6.3 | 0.801 |
| Percent of predicted VO_2_max, % | 56 ± 13 | 54 ± 12 | 56 ± 14 | 0.799 |
| Percent of predicted maximal heart rate, % | 85 ± 14 | 75 ± 15 | 86 ± 13 | 0.059 |
| Heart rate reserve, bpm | 89 ± 31 | 68 ± 31 | 93 ± 29 | 0.063 |
| Heart rate recovery, bpm | 21 ± 9 | 19 ± 15 | 22 ± 8 | 0.767 |
| Chronotropic index | 0.80 [0.54-0.87] | 0.47 [0.41-0.83] | 0.82 [0.69-0.87] | 0.099 |
| Oxygen pulse, mL/beat | 9.3 [7.8-10.3] | 10.2 [8.1-11.9] | 9.3 [7.8-10.0] | 0.467 |

All data is shown as median [Q1-Q3], mean ± SD, or n (%).

LF: low frequency power, HF: high frequency power, Q: quartile, QTc: corrected QT interval according to Bazett, rMSSD: square root of the mean squared differences of successive NN intervals, SD: standard deviation, SDNN: standard deviation of all NN intervals, SDANN: standard deviation of the average NN intervals calculated over 5-minute intervals, pNN50: percentage of adjacent NN intervals that differ by>50 ms, VLF: very low frequency power.
